# Improved diagnostics targeting c-MET in non-small cell lung cancer: expression, amplification and activation?

**DOI:** 10.1186/s13000-015-0362-5

**Published:** 2015-07-28

**Authors:** I. Watermann, B. Schmitt, F. Stellmacher, J. Müller, R. Gaber, Ch. Kugler, N. Reinmuth, R. M. Huber, M. Thomas, P. Zabel, K. F. Rabe, D. Jonigk, A. Warth, E. Vollmer, M. Reck, T. Goldmann

**Affiliations:** Clinical and Experimental Pathology, Research Center Borstel, Borstel, Germany; LungenClinic Grosshansdorf, Grosshansdorf, Germany; Airway Research Center North (ARCN), Member of the German Center for Lung Research (DZL), Borstel, Germany; Ludwig Maximilians University (LMU), Munich, Germany; Comprehensive Pneumology Center Munich, (CPC-M), Member of the German Center for Lung Research, Thoracic Oncology Centre Munich, Munich, Germany; Medical Clinic, Research Center Borstel, Borstel, Germany; Institute of Pathology, Hannover Medical School, Hanover, Germany; Biomedical Research in Endstage and Obstructive Lung Disease Hanover (BREATH), Member of the German Center for Lung Research, Munich, Germany; Institute of Pathology, Heidelberg University, Heidelberg, Germany; Translational Lung Research Center (TLRC), Member of the German Center for Lung Research, Heidelberg, Germany; Pathology, Faculty of Medicine, Alexandria University, Alexandria, Egypt

**Keywords:** Non-small cell lung Cancer (NSCLC), MET, Targeted therapies, Diagnostic, Phosphorylated MET, Immunohistochemistry, Fluorescence in situ hybridization

## Abstract

**Background:**

Several c-MET targeting inhibitory molecules have already shown promising results in the treatment of patients with Non-small Cell Lung Cancer (NSCLC). Combination of EGFR- and c-MET-specific molecules may overcome EGFR tyrosine kinase inhibitor (TKI) resistance. The aim of this study was to allow for the identification of patients who might benefit from TKI treatments targeting MET and to narrow in on the diagnostic assessment of MET.

**Methods:**

222 tumor tissues of patients with NSCLC were analyzed concerning c-MET expression and activation in terms of phosphorylation (Y1234/1235 and Y1349) using a microarray format employing immunohistochemistry (IHC). Furthermore, protein expression and MET activation was correlated with the amplification status by Fluorescence in Situ Hybridization (FISH).

**Results:**

Correlation was observed between phosphorylation of c-MET at Y1234/1235 and Y1349 (spearman correlation coefficient r_s_ = 0.41; p < 0.0001). No significant correlation was shown between MET expression and phosphorylation (p > 0.05). c-MET gene amplification was detected in eight of 214 patients (3.7 %). No significant association was observed between c-MET amplification, c-MET protein expression and phosphorylation.

**Conclusion:**

Our data indicate, that neither expression of c-MET nor the gene amplification status might be the best way to select patients for MET targeting therapies, since no correlation with the activation status of MET was observed. We propose to take into account analyzing the phosphorylation status of MET by IHC to select patients for MET targeting therapies. Signaling of the receptor and the activation of downstream molecules might be more crucial for the benefit of therapeutics targeting MET receptor tyrosine kinases than expression levels alone.

## Background

Targeted therapies constitute promising strategies in the personalized treatment of cancer. Increasing knowledge of expression patterns and molecular pathophysiology led to improved outcomes of patients with non-small cell lung cancer (NSCLC). The Mesenchymal Epithelial Transition factor (MET) receptor tyrosine kinase (RTK) and its ligand hepatocyte growth factor/scatter factor (HGF/SF) are predominantly involved in epithelial-to-mesenchymal transition (EMT) [1] and tissue regeneration [2]. Binding of HGF induces MET dimerization that induces tyrosine kinase activation by phosphorylation of the tyrosine residues Y1230, Y1234 and Y1235, which then activates various downstream signaling cascades [3]. Activated MET receptor signaling promotes tumor angiogenesis, tumor cell invasion and metastasis [4, 5]. This constitutive activation is thought to be due to MET overexpression, gene amplification and mutations within the tyrosine kinase domain and correlates with poor clinical outcome in patients with lung cancer [3, 6–10].

Several MET-targeting inhibitors have already shown promising data in clinical trials [11–13]. Most of them are tyrosine kinase inhibitors followed by antagonistic antibodies [14, 15]. Onartuzumab, a recombinant, fully humanized monovalent, monoclonal antibody binds to the extracellular domain of MET, thus blocking binding of HGF and thereby the activation [16, 17]. Spigel et al. conducted a randomized phase II trial of onartuzumab in combination with erlotinib in patients with advanced NSCLC [18]. Onartuzumab plus erlotinib was associated with improved progression-free survival (PFS) and overall survival (OS) in the MET-positive population. Nevertheless, the phase III clinical study for this antibody was stopped because the results of phase II could not be confirmed. The onartuzumab/erlotinib combo did not show an overall survival benefit for the patients, even in the high expressers of MET [19].

Tivantinib, a c-MET selective, small molecule inhibits MET phosphorylation leading to reduced capacity of invasion, proliferation and metastasis [20, 21]. Results from the MARQUEE trial corroborate an OS benefit for patients with locally advanced or metastatic non-squamous MET-high lung cancer who received chemotherapy prior to treatment with tivantinib in combination with erlotinib. Unfortunately, the primary end point or improved OS for the whole group was not met.

Crizotinib, a non-selective MET inhibitor targeting c-MET, ALK and ROS1 exhibits its antitumoral effect in c-MET, ALK-positive and ROS positive patients [11, 22, 23] demonstrating the need of MET determination. The expression of MET was not evaluated in these studies. In addition, concurrent inhibition of VEGFR2 and MET is discussed for anti-angiogenesis therapy, also for NSCLC [24].

MET amplification leads to gefitinib resistance in lung cancer patients lacking the point mutation T790M in exon 20 of EGFR [6]. Bean et al. have also shown that MET amplification occurs in human lung adenocarcinomas independently of T790M with resistance to gefitinib or erlotinib, emphasizing the relevant therapeutic target of MET for patients with acquired resistance to EGFR kinase inhibitors. Combination of EGFR and MET inhibitory molecules may overcome EGFR TKI resistance in patients with NSCLC [6]. MET as target of several multi TKIs, together with the prognostic value of MET expression demonstrating the relevance to evaluate MET on several levels. The challenge is to identify patient subgroups who respond to MET inhibitors. For that reason the main goal is to establish a reliable predictive assay for the determination of the MET status.

For that purpose, we investigated in a retrospective study, MET expression on protein level by IHC using antibody clone SP44 in 222 formalin fixed paraffin embedded (FFPE) - and HOPE (HEPES-Glutamic Acid Buffer Mediated Organic Solvent Protection Effect) fixed - NSCLC tissues. Because not only expression but also activation of MET is essential for tumor progression, we also analyzed the phosphorylation status of the receptor by IHC with two phosphorylation specific antibodies. To obtain more insights into the relation between MET expression, phosphorylation and amplification, MET amplification was assessed by FISH analysis. For a limited number of tumor samples, MET expression was also evaluated on mRNA level and compared with MET expression evaluated by Western Blot analysis.

Finally, we compared MET- and EGFR-expression within these tumor tissues to investigate, whether a correlation exists between both biomarkers. The aim of the present study was to assess, if the expression and amplification is correlated with the activation status of the MET receptor, because we hypothesize, that not only expression and amplification but phosphorylation can be a relevant marker to select patients for MET specific therapies.

## Methods

### Patients and tissues

A total of 222 lung tumor specimens from patients with NSCLC were obtained from the Biomaterial Bank North after resection from the surgical department of LungenClinic Grosshansdorf (Table 1), including 110 cases of adenocarcinoma (ADC), 86 cases of squamous cell carcinoma (SCC), 12 cases of large cell carcinoma (LCC), 6 cases of carcinoids and 2 cases of adeno-squamous carcinoma. This retrospective study was performed in compliance with the ethical committee of the University of Lübeck (reference number 12–220). All tumor samples were histologically classified according to the International Association for the Study of Lung Cancer/American Thoracic Society/European Respiratory Society International Multidisciplinary classification of lung adenocarcinoma 2011 [25] and WHO guidelines 2010 [26]. The lung cancer tissue samples were fixed both with formalin and in parallel using the alternative HOPE technique and were subsequently embedded in paraffin [27]. Established clinical and histological factors of all tested patients were included in this study (patient age, gender, histology, TNM classification, smoking status; Table 1).

**Table 1:** Characteristics of 222 patients with Non-small cell lung cancer

Category	Subcategory	Results (%)
Age	≥65	154 (69.4 %)
	<65	68 (30.6 %)
Gender	Male	142 (63.9 %)
	Female	80 (36.1 %)
^a^Smoking status	Current	67 (30.1 %)
	Former	101 (45.5 %)
	Never	23 (10.4 %)
	unknown	31 (14 %)
^a^Asbestos contact	Present	17 (7.7 %)
	Absent	49 (22.1 %)
^a^COPD	Present	43 (15.3 %)
	Absent	5 (2.3 %)
Histologic type	ADC	110 (49.5 %)
	SCC	94 (42.3 %)
	LCC	9 (4.1 %)
	Other	9 (4.1 %)
Tumor size	T1	43 (19.4 %)
	T2	125 (56.3 %)
	T3	32 (14,4 %)
	T4	22 (9.9 %)
Lymph node status	N0	106 (47.7 %)
	N1	52 (23.4 %)
	N2	47 (21.2 %)
	N3	17 (7.7 %)
Stage	I	72 (32.4 %)
	II	61 (27.5 %)
	III	79 (35.6 %)
	IV	10 (4.5 %)

^a^History of smoking, contact with asbestos and chronic obstructive pulmonary disease (COPD) were undetermined in the rest of the patients

### Tissue microarray (TMAs) construction

Specimens were arranged on TMAs for enhanced comparability of immunohistochemical stainings as previously described [28]. Appropriate formalin and HOPE-fixed -paraffin embedded A549 cell line known to express c-MET basal, was used as control. Representative tumor punches (2 mm in diameter) of two core biopsies were taken from two viable parts of each tumor block using the Beecher manual arrayer (Beecher instruments, Alpha, Metrix Biotech). This approach should enhance representative analyzing of immunohistochemical stainings and FISH analyzes.

### Immunohistochemistry (IHC)

MET protein expression was assessed by IHC on 2 μm thick deparaffinized TMA sections, using rabbit anti-human c-Met monoclonal antibody (clone SP44) (Spring Biosciences, Pleasanton, CA, USA) directed against the synthetic peptide derived from C-terminus of human c-Met protein displaying membranous and/or cytoplasmic epitope. Staining procedures were conducted according to manufacturer’s protocol in a final 1:1000 dilution.

Phosphorylated MET-expression was assessed by two different primary antibodies purchased from Cell Signaling (Danvers, MA). Phospho-Met (Y1234/1235) (D26) and Phospho-Met (Y1349) (130H2) in a final dilution of 1:100.

HOPE-fixed, paraffin-embedded sections were deparaffinized by xylene incubation (two times 10 min) before SP44 was applied for 30 min at room temperature. Endogenous peroxidases were blocked by incubation with 3 % H_2_O_2_ for 10 min. Negative controls omitting the primary antibody were always included.

Formalin-fixed samples from the same tumors were deparaffinized following standard procedure. Antigen retrieval was achieved by boiling in citrate buffer (10 mM, pH 6.0) for 10 min (Merck KGaA, Darmstadt, Germany) with subsequent cooling at room temperature for 20 min. Antigen detection was carried out with DAB substrate kit (DAB chromogen and DAB substrate), used for 15 min to visualize specific binding. Sections were counterstained using Meyer’s hemalum and mounted with Pertex (Medite GmbH, Burgdorf, Germany) for further evaluation.

### Evaluation of Met IHC staining

Two observers quantified staining intensities. Samples were evaluated with the MET scoring algorithm as previously described [18]. MET positivity was defined when scored as 2+ and 3+. 3+ was selected if ≥ 50 % of tumor cells stained with strong intensity; 2+ was defined if ≥ 50 % of tumor cells showed moderate or higher staining but < 50 % with strong intensity; 1+ if ≥ 50 % of tumor cells with weak or higher staining but < 50 % with moderate or higher intensity; 0 was defined if no staining or <50 % of tumor cells with any intensity.

Phosphorylated MET expression was evaluated according to the intensity of cytoplasmic staining (no staining = 0, weak staining = 1, moderate staining = 2, strong staining = 3) and the percentage of stained tumor cells (0 % = 0, 1-10 % = 1, 11-50 % = 2, >50 % = 3). If ≥ 50 % of tumor cells showed staining intensity ≥ 2, tumor samples were categorized as positive in this study.

### EGFR scoring methodology

EGFR expression is considered as positive, if ≥10 % of the tumor cells show membranous staining of any intensity using x10 and x20 magnification assessed by Dako EGFR PharmDx data sheet.

### Fluorescence in situ hybridization

c-MET gene copy numbers were assessed by fluorescence in situ hybridization (FISH) using ZytoLight SPEC MET/CEN7 Dual Color Probe (Zytovision, Bremerhafen, Germany). Before hybridization, sections were deparaffinized, dehydrated and immersed in citrate buffer (Merck KGaA, Darmstadt, Germany) pH 6 at 98 °C for 15 min, and subsequently washed twice in distilled water for 2 min. The sections were air dried and pretreated with pepsin for 5 min before denatured for 10 min at 75 °C. After hybridization at 37 °C for 20 h, slides were washed and counterstained with 1.5 μg/ml 4’,6’-diamidino-2-phenylindole (DAPI) mounting medium (Vectashield, Vector laboratories, Burlingame, CA) and coverslips were fixed with nail polish.

Analysis of FISH signals was performed on an epifluorescence microscope Nikon Eclipse 80i H550L (Nikon) with interference filters (AHF Analysentechnik AG, Tübingen, Germany).

At least 50 non-overlapping interphase nuclei of tumor cells were counted per core. The nuclei were selected using the DAPI filter under high magnification (x600). For each probe, the numbers of c-MET and CEN7 per nuclei were separately scored and mean cMET/CEN7 ratio was determined. FISH MET gene amplification was defined as FISH positive, when

- MET/CEP7 ratio was > 2.2 or
- small gene clusters (≥4 copies) independent of the MET to CEP 7 ratio

These analysis criteria were adapted to previous publications for the evaluation of MET amplification [7].

Increased MET gene copy number (GCN) >3 of Chromosome 7 and MET-locus was defined as polysomy.

Evaluation was performed independently by two scientists well-vised in FISH analysis.

### Protein extraction, SDS-PAGE, and western blotting

HOPE-fixed, paraffin-embedded specimens were deparaffinized as previously described by Olert et al. [27]. Protein extraction was performed using lysis buffer (7 M urea, 2 M thiourea, 100 mM dithiothreitol (DTT), 4 % CHAPS (3-[3-Cholamidopropyl)-dimethyl-ammonio]-1-propansulfonat), 2 % IGEPAL, 1 % Triton X, 5 mM PMSF, 0.5 mM EDTA, 40 mM Tris). Protein concentration was determined by Bradford with Coomassie (Bradford) Protein Assay Kit (Pierce, Rockford, IL, USA). Forty μg of whole protein lysate was separated by gel electrophoresis and analyzed by Western Blot using nitrocellulose membrane using iblot gel transfer system (Invitrogen, Germany). After blocking with 5 % nonfat dry milk (1 h, ambient temperature), membranes were incubated with Met-specific antibody clone SP44 (1:1000) in TBS-0.05 % Tween overnight (4 °C). The following day, the membrane was washed three times in TBS-0.05 % Tween before adding secondary antibody anti rabbit HRP (Cell Signaling) for 1 h at room temperature. Development was achieved using ECL solution and exposure to film. Intensities of appropriate MET specific bands were analyzed by band leader.

### Quantitative real-time PCR of MET mRNA

Total RNA was isolated from HOPE-fixed tissue samples as described previously [29] using RNeasy minikit (Qiagen) according to the manufactures protocol. RNA integrity and concentration were determined with the Agilent RNA 6000 Nano Assay Bioanalyzer (Agilent, Böblingen, Germany). 1 μg of RNA was transcribed into cDNA with the Maxima first strand cDNA synthesis kit for RT-qPCR (Thermos Scientific). Genomic DNA was digested with DNase I within cDNA synthesis. RPL32 was used to normalize differences in input cDNA amounts. Intron spanning primers were designed with the Universal Probe Library (Roche Applied Sciences, Germany). Primers were used as follows: Forward primer 5’-TGAAATTCATCCAACCAAATCTT-3’ and reverse primer 5’- AATAGAAAACTGACAATGTTGAGAGG-3’ (probe no. 31) for MET and forward primer 5’- CCACCGTCCCTTCTCTCTT-3’ and reverse primer 5’- GGGCTTCACAAGGGGTCT-3’ (probe no. 10) for RPL32. Amplification was performed with Light Cycler 480II from Roche Diagnostics. After initial denaturation at 95 °C for 5 min, amplification protocol consisted of 45 cycles (10 s at 95 °C and 30 s at 60 °C) using Light Cycler 2x Probe Master Mix, 0.4 μM oligonucleotides and 0.2uM 6-carboxyfluorescein (FAM)-labeled hydrolysis probes (Roche Applied Science) in a final volume of 10 μl. Standard curve was made from serial dilution of template cDNA. Expression levels were calculated after normalization with RPL32.

### Statistical analysis

All statistical analysis in this study were performed with Spearman’s rank nonparametric correlation test using Prism 6 software. All reported p values are two sided (GraphPad Prism version 6.0 Software, San Diego, CA).

## Results

### Patient characteristics

A total of 222 surgically resected NSCLC patients were included in this study. Clinical parameters are summarized in Table 1. 110 tumors (49.5 %) were classified as adenocarcinoma (ADC), 94 (42.3 %) were grouped as squamous cell carcinoma (SCC), 9 (4.1 %) as large cell carcinoma (LCC) and 9 (4.1 %) as other tumors (typical and atypical carcinoids). According to WHO classification 2010, pathological TNM staging distributed the patients as followed: 29 (13 %) of patients were classified as IA, 43 (19.4 %) were classified as IB 40 (18 %) were IIA, 21 (9.5 %) IIB, 56 (25.2 %) were IIIA, 23 (10.4 %) were classified as IIIB and 10 (4.5 %) were grouped to IV (Table 1).

### MET protein expression evaluated by IHC in FFPE- versus HOPE fixed NSCLC tissues

**Fig. 1:**
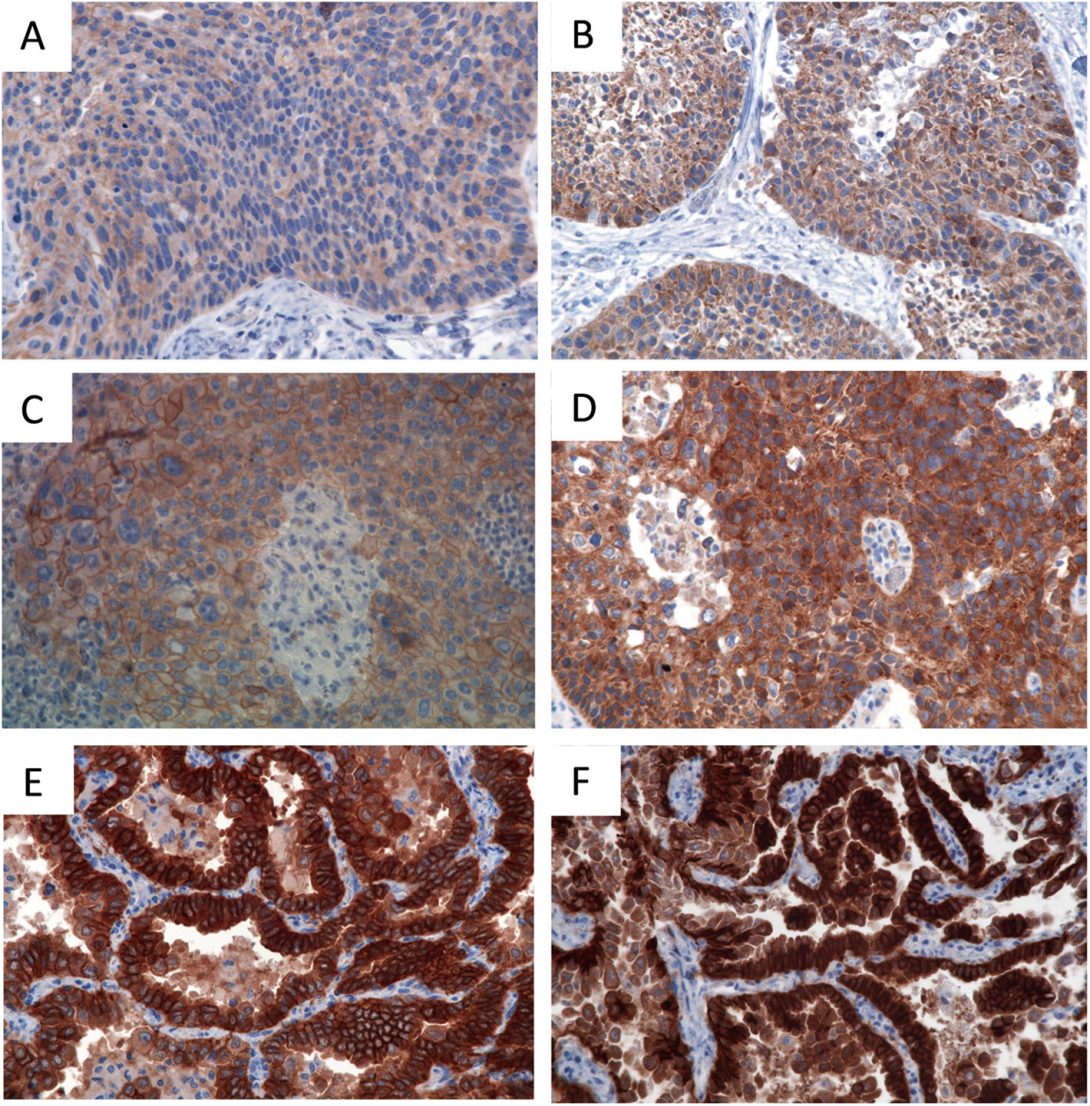
Examples of NSCLC tissues stained for immunohistochemical quantification of MET differences between FFPE- and HOPE-fixed tumor samples, stained with MET specific antibody clone SP44 (original magnification x 400). a + b) same specimen. **a** FFPE: MET expression scored 0; **b** HOPE fixed sample; MET expression scored 1+. (c + d) one specimen. **c** FFPE: MET expression scored 1+; **d** HOPE fixed sample; MET expression scored 2+. (e + f) one specimen. **e** FFPE: MET expression scored 2+; **f** HOPE fixed sample; MET expression scored 3+

We compared staining intensities of the MET specific antibody clone SP44 in formalin fixed paraffin embedded tissue versus HOPE-fixed tumor samples. When MET expression was evaluated in HOPE-fixed samples, staining intensities were mostly stronger compared to the appropriate FFPE tissue samples within one specimen (Fig. 1). In FFPE NSCLC tissues, 209 samples could be analyzed concerning their staining intensities. 45 (21.5 %) were scored as MET positive (2+). None of the FFPE tissues showed cMET expression of 3+. Negative MET expression was observed for 164 (78.5 %) tumor samples 53 (25.4 %) cases were scored as 0, and 111 (53.1 %) tumor samples were scored as 1). Of the ADC subtype, 34 (16.2 %) were scored as MET positive, of the SCC subtype 10 (4.8 %) and of the LCC 1 (0.5 %) (Table 2).

**Table 2:** Relationship between histological entities and MET protein expression and gene copy number in 209 patients with NSCLC

Histology versus MET expression (formalin)			
	All	MET positive	MET negative
		(formalin)	(formalin)
Histology (%)	209 (94.1)	45 (21.5)	164 (78.5)
ADC	110 (48.2)	34 (16.2)	71 (34)
SCC	94 (42.3)	10 (4.8)	77 (36.8)
LCC	9 (4)	1 (0.5)	8 (3.8)
other	9 (4)	0	8 (3.8)
Histology versus MET expression (HOPE)			
	All	MET positive	MET negative
		(HOPE)	(HOPE)
Histology (%)	209 (94.1)	77 (36.8)	132 (63.2)
ADC	110 (48.2)	54 (25.8)	51 (24.4)
SCC	94 (42.3)	23 (11)	64 (30.6)
LCC	9 (4)	0 (0)	9 (4.3)
other	9 (4)	0 (0)	8 (3.8)
Histology versus MET phosphorylation at Y1234/1235			
	All	Y1234/1235+	Y1234/1235-
Histology (%)	209 (94.1)	30 (14.4)	179 (85.6)
ADC	110 (48.2)	20 (9.6)	87 (41.6)
SCC	94 (42.3)	9 (4.3)	77 (36.9)
LCC	9 (4)	1 (0.5)	7 (3.3)
other	9 (4)	0	8 (3.8)
Histology versus MET phosphorylation at Y1349			
	All	Y1349+	Y1349-
Histology (%)	209 (94.1)	38 (18.1)	171 (81.8)
ADC	110 (48.2)	17 (8.1)	91 (43.5)
SCC	94 (42.3)	18 (8.6)	67 (32.1)
LCC	9 (4)	3 (1.4)	5 (2.4)
other	9 (4)	0	8 (3.8)
Histology versus MET gene copy number			
	All	FISH positive	FISH positive
		(amplification)	(polysomy)
Histology (%)	209 (94.1)	8 (3.8)	5 (2.4)
ADC	110 (48.2)	5 (2.4)	3 (1.4)
SCC	94 (42.3)	3 (1.4)	0 (0)
LCC	9 (4)	0 (0)	2 (1)
other	9 (4)	0 (0)	0 (0)

**Fig. 2:**
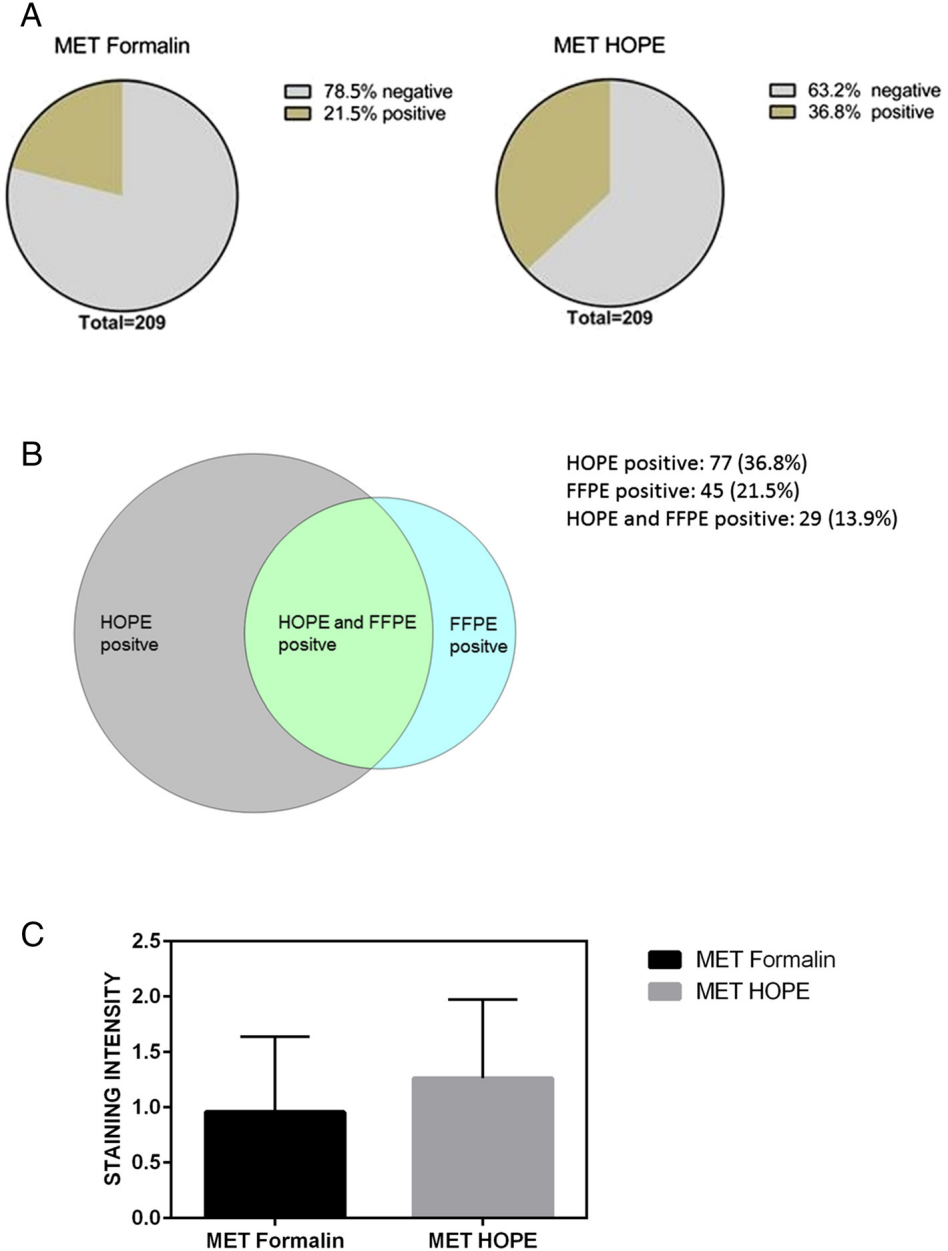
**a** Distribution of positive and negative MET expression of NSCLC samples. **b** Intersection of MET positive Formalin- and HOPE- fixed NSCLC samples. **c** Mean of MET intensities of Formalin- versus HOPE- fixed NSCLC samples

Of the corresponding 209 HOPE fixed NSCLC tissues, 77 (36.8 %) were scored positive (72 (34.4 %) of the tumor samples were scored as 2+ and 5 (2.4 %) were classified as 3+). Negative MET expression was shown for 132 (63.2 %) tumor tissue samples (28 (13.4 %) samples were scored as 0 and 104 (49.8 %) samples were scored as 1) (Fig. 2a). Subtype classification of HOPE fixed tumor yielded in 54 (25.8 %) MET positivity for ADC, 23 (11 %) for SCC, none of LCC and other subtypes showed MET expression (Table 2).

There was no significant association with clinical characteristics and also not with the histological entities (data not shown). Of 77 (36.8 %) HOPE positive samples and 45 (21.5 %) FFPE- positive samples, the intersection between HOPE and formalin fixed tumor tissue was 29 (13.9 %) (Fig. 2b). Mean MET IHC score was 0.962 (SD 0.685) for FFPE tumor tissue samples and 1.258 (SD 0.714) for the appropriate HOPE fixed samples (Fig. 2c).

Correlation between c-Met expression evaluated by IHC in FFPE versus HOPE fixed samples was shown (spearman correlation coefficient r_s_ = 0.482 p < 0.0001). Correlation with any clinicopathological status was not shown.

### Expression of activated MET [Y1234/1235] and [Y1349]; correlation between MET expression and phosphorylation

Of 222 patients, 213 tumor samples could be evaluated for phosphorylation at Y1234/1235 (autophosphorylation site). 30 (14 %) tumor tissue samples showed Met phosphorylation. Of 209 tumor tissues, evaluated by IHC for the expression of MET and positive for phosphorylation at Y1234/1235, 20 (9.6 %) were classified as ADC, 9 (4.3 %) were categorized as SCC and 1 (0.5 %) as LCC. For phosphorylation at Y1349 (docking site of MET), 215 tissues could be analyzed. 38 (17.7 %) samples were phosphorylated at that phosphorylation site. Classified into the corresponding histological entities, MET phosphorylation of Y1349 was present in 17 (8.1 %) of ADC, 18 (8.6 %) of SSC and 3 (1.4 %) of LCC related to the 209 tissues evaluated for MET expression (Table 2).

**Fig. 3:**
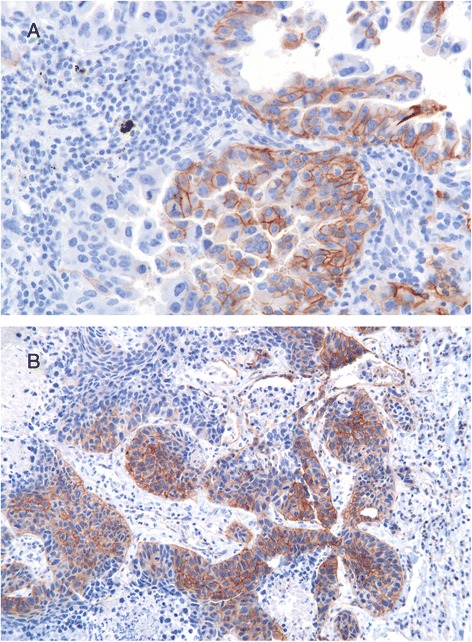
Examples for immunohistochemical Analysis of activated p-MET [Y1234/1235] and [Y1349] in NSCLC tumor tissues (original magnification x 400). **a** Phosphorylation of Y1234/1235. **b** Phosphorylation of Y1349

Correlation between phosphorylation of MET at Y1234/1235 and Y1349 was determined with r_s_ = 0.41; p < 0.0001). Staining patterns for phosphorylated MET (Y1234/1235 and Y1349) were both cytoplasmic and membranous. Immunoreactivity was exclusively restricted to tumor cells (Fig. 3). Next, correlation between phosphorylated MET and expression was evaluated. For that purpose, expression and phosphorylation were categorized into positive and negative as described above. Relationship between MET expression and phosphorylation was unverifiable. Correlation between MET expression and phosphorylation was neither shown for Y1234/1235 nor for Y1349. (spearman correlation coefficient r_s_ = 0.054, p > 0.05 for Y1234/1235 and MET expression and r_s_ = 0.017, p > 0.05 for Y1349 and MET expression). Likewise, no association with any clinicopathological parameter was shown.

**Fig. 4:**
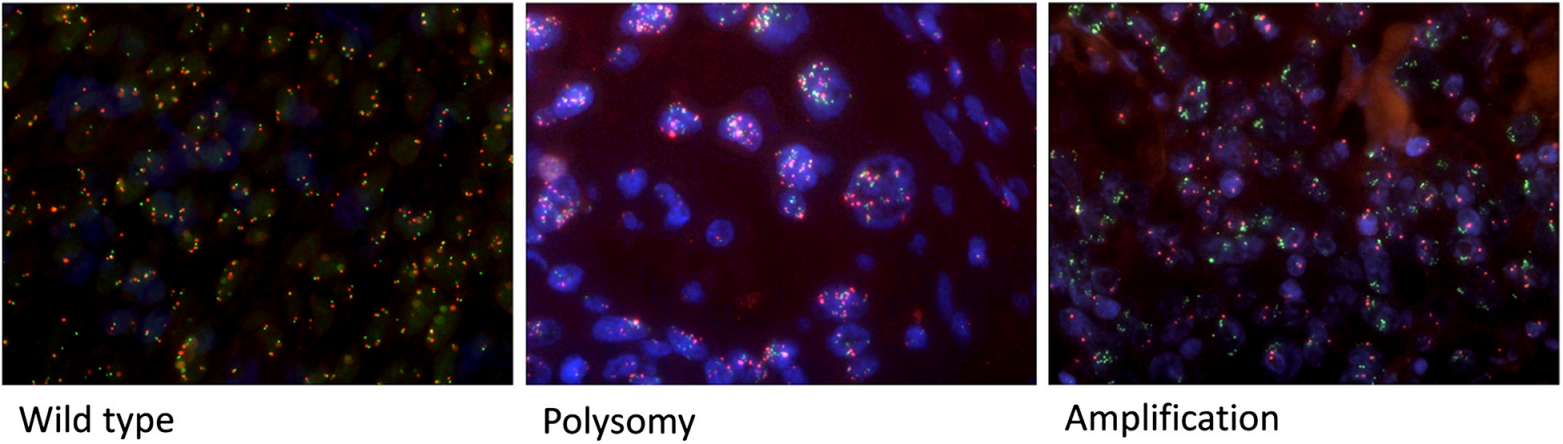
MET gene copy numbers evaluated by FISH analysis

### MET gene copy numbers evaluated by FISH

Out of total of 444 tumor samples (222 patients in duplicates), valid results were obtained for 214 patients (96.4 %). Invalid results were due to the lack of adequate tumor cells.

C-MET gene amplification was detected in eight of 214 patients (3.7 %). Concerning the 209 tumor tissues evaluated for MET expression, percentage acounts 3.8 %. These clustered gene amplifications showed mean ratio range between 1.5 and 6.26 (cMET/CEN7 ratio), distributed in 5 (2.4 %) of ADC and 3 (1.4 %) of SCC whereas no amplification was observed in LCC and other tumor entities (Table 2). Polysomies were evident in 2.3 % of analyzed tumor samples (corresponds to 2.4 % of 209 tumor samples analyzed for MET expression). Thereof, 3 (1.4 %) were ADC, and 2 (1 %) were LCC, no polysomies were observed in SCC and other tumor entities (Table 2). Different FISH patterns are shown in Figure 4.

### Correlation between MET amplification, MET protein expression, and activation

No significant association was observed between c-MET amplification and MET protein expression or phosphorylation (MET amplification and MET expression Formalin: spearman correlation coefficient r_s_ =0.06, p > 0.05; MET amplification and MET expression HOPE r_s_ <0, p > 0.05; MET amplification and phosphorylation at Y1234/1235: spearman correlation coefficient r_s_ =0.089, p > 0.05; amplification and phosphorylation at Y1349: spearman correlation coefficient r_s_ =0.052, p > 0.05).

MET gene copy number correlated with tumor size by trend and lymph node status (both: spearman correlation coefficient r_s_ = 0.132; p = 0.012). No association was found for any other clinical characteristic. (MET amplification is composed of real amplification plus polysomies).

### Quantification of MET by IHC, western blot analysis and real-time PCR (mRNA-level)

#### Relationship between MET determination on mRNA-level, protein level, DNA-level and the activated MET receptor

**Fig. 5:**
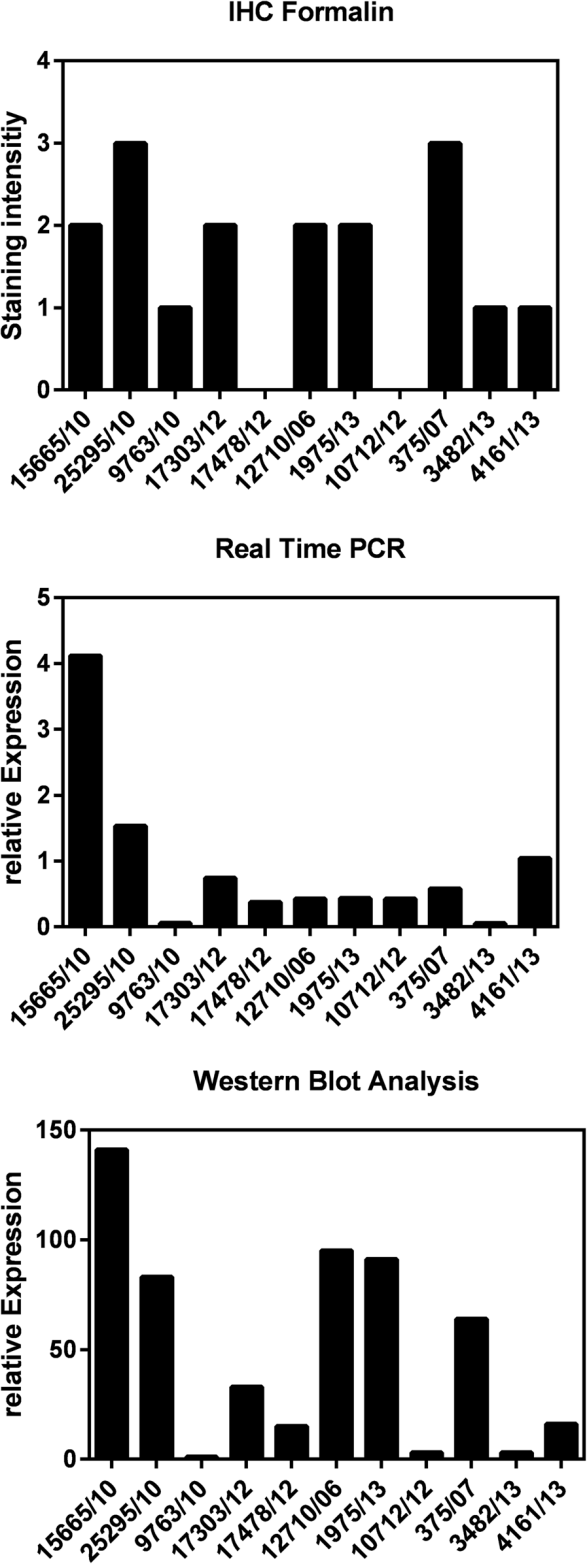
Quantification of MET. Comparison of IHC, Western Blot Analysis and Real-Time PCR

A few NSCLC tissue samples (11 tumor samples are shown) could be selected for the comparison of MET expression levels evaluated on protein level by IHC and western blot analysis and on mRNA-level by real-time PCR. Strong relationship is observed between IHC and western blot analysis (Fig. 5). Whereas no explicit connection is found between the expression on protein level and mRNA level, independently of evaluating the protein level of MET by IHC or western blot analysis (Fig. 5).

#### Correlation between EGFR expression and MET expression evaluated by IHC

In a previous publication, we analyzed EGFR expression by different EGFR specific antibody clones [30]. Therefore, we were interested in the relationship between MET – and EGFR- expression. Correlation between MET-and EGFR-expression, evaluated with antibody clone 31G7, was determined. (Spearman correlation coefficient r_s_ = 0.2086; p = 0.0026). 207 tumor tissues could be analyzed for both MET- and EGFR-expression. 185 tumor samples were scored as positive for the expression of EGFR, 44 were scored positive for MET expression and 43 tumor tissues were double positive for EGFR and MET. Thus, nearly all of MET expressing tumor samples were also positive for MET expression.

## Discussion

Personalized therapies will only be effective if target molecules are exactly determined or quantified. MET is currently one of the target proteins that are utilized for several anticancer therapies in patients with NSCLC [14, 15, 31]. State of the art methods for the determination of MET have been previously conducted by IHC and FISH-analysis. Within the present study, we show for the first time combined analysis of MET expression on protein-, mRNA-, and DNA-level together with the investigation of activated MET RTK by immunohistochemistry. We demonostrated that in resected specimens no correlation exists between MET-expression and -phosphorylation. In fact, activated MET is a potential target for small molecule tyrosine kinase inhibitors that already have entered clinical trials.

Dziadziuszko et al. [32] demonstrated a significant correlation between MET gene copy number determined by SISH and protein expression evaluated by IHC. In the METLung clinical trial [18] of Spiegel et al., IHC was assessed using antibody clone SP44 for the determination of MET-expression. Patients with 2+ or 3+ MET expression of their tumours were included. They were stratified for the level of MET expression. There was no improvement in PFS or OS in the Intention-To-Treat (ITT) population. Expression of c-MET was associated with worse outcome. Apparently, the addition of onartuzumab to erlotinib in MET-positive patients abrogates the negative prognostic impact. But, without a diagnostic hypothesis, the results obtained in the (ITT) population lead to interrupt the development of onartuzumab. One reason for the weak responsiveness might be that not only the expression level of c-met is relevant, but also the status of receptor signaling.

In the trial of Spigel et al. [33] 52 % of patients were scored as MET positive compared to 25 % in the study of Dziadziuszko et al. [32] and 21.1 % of FFPE tumor tissue versus 36.8 % HOPE fixed tumor samples in our population. The high number of MET IHC positivity is estimated to the fact, that in the METLung trial patients with advanced NSCLC were analyzed. The difference between FFPE and HOPE fixed tumor samples might be explained by the assumption that HOPE fixed tissues generally reflect a more de facto protein expression as a consequence of improved preservation of antigenicity [34]. Nevertheless, a correlation between c-Met expression of FFPE and HOPE-fixed samples was shown. The intersection of FFPE- and HOPE fixed tissues accounted 13.9 %, demonstrating, that the majority of FFPE tissues is included in the HOPE-fixed c-MET positive determined group of samples. We did not observe any correlation with the clinicopathological status of the patients, even not with the histologic types and stage, independently of the fixation method.

There are many studies evaluating the expression level of c-MET, but only few that investigated phosphorylated c-MET in human cancer tissues. Whether MET-expression is correlated with its phosphorylation status in NSCLC tissue, still remains unclear. Recently, in the case of gastric cancer, Inoue et al. [35] have shown, that expression of c-MET and its phosphorylation are not always correlated. Whereas, Nakamura et al. [36], observed overexpression of c-MET in 74.6 % of 130 surgically resected ADC, phosphorylation of Y1235 was detected in 21.5 %. In addition, phosphorylated c-MET was correlated with high levels c-MET expression (P = 0.0303) [36].

In our study, MET phosphorylation at Y1234/1235 was detected in 14 % of the investigated NSCLC tumor samples, phosphorylation of Y1349 was observed in 17.7 %. No association between MET expression and phosphorylation was found. In contrast to the study of Nakamura et al., differences might be due to distinct statistical analysis. Nakamura et al., determined association with chi-square test, whereas in our study, correlation was computed with spearman rank correlation. Another reason could be the application of different antibody clones for activated MET Y1234/1235 compared to the study of Nakamura et al. phosphorylation at [Y1349] has not been investigated by Nakamura et al. [36].

Unfortunately, association analysis between the expression and phosphorylation of MET with clinical characteristics of the patients was not possible, because we do not know anything about course of disease, e.g. disease progression or treatment with TKIs.

Several studies have already been addressed to evaluate the MET amplification status [7, 32, 37] by FISH. But, previous studies used different criteria to define MET amplification [7, 37, 38] in NSCLC. MET amplification has been detected in up to ~5 % [36, 39, 40], whereas an increased MET gene copy number was counted in ~20 % of patients with NSCLC by PCR based technique [37, 41–43]. Inconsistence might be due to the genetic alterations: gene amplification and polysomy. Bean et al. determined MET gene amplification in 3 % of untreated patients [37]. Cappuzzo et al. observed true gene amplification in 4.1 % of 447 NSCLC patients [7]. In our study, frequency of met gene amplification is 3.7 % matching with the results of both referred reports given [7, 37]. Polysomy was detected in 2.3 % of the investigated tumor samples. Correlation of MET gene amplification also including polysomies showed no association with MET protein expression. These results are contrary to the study of Dziadziuszko et al., who found significant correlation between MET gene copy number and MET protein expression [32]. One reason for that discrepancy could be due to different IHC scoring systems, Dziadziuszko et al. did the evaluation of IHC by using the H-score assessment. Another difference to our study is the use of SISH technology by Dziadziuszko et al. compared to the FISH technique we were conducting. Quantification of MET by IHC, western blot analysis and real time PCR was performed for a subset of NSCLC tumor samples to figure out an idea of whether there is any connection between these three quantification methods. Strong correlation was observed between IHC evaluation of MET protein expression and western blot analysis. Apparently, no clear relationship exists between the expression on mRNA - and protein level. Expression of MET on mRNA level seems to be less pronounced than on protein level (Fig. 5). These differences could be explained by posttranslational modifications or degradation.

Unfortunately, we have almost no data about the therapeutic management and the course of disease.

Finally, we have shown that there is a tendency of association between MET and EGFR expression. Almost tumor samples expressing MET are also positive for EGFR expression. Cross-talk between MET and EGFR in NSCLC has been previously shown by Acunzo et al. [44]. These data are relevant to interpret the results of Benedettini et al. [45]. In this study, MET protein expression and phosphorylation have been associated with primary resistance to EGFR-TKIs in patients with NSCLC. Also Bean and colleagues observed MET amplification in 21 % of patients with acquired resistance to EGFR-TKIs demonstrating the crosstalk between both receptor signaling pathways [37]. Thus, there could be a direct relationship between co-expression of MET and EGFR and the responsiveness to EGFR-and or MET-targeting TKI. Data are published showing that EGFR signaling is sufficient to induce the phosphorylation of MET [46].

## Conclusion

Our study depicts, that definition of MET positive signaling together with MET-positivity might be required to predict the benefit of MET targeting therapies. We hypothesize that not MET expression is crucial for efficacy of MET inhibitory molecules but the evaluation of the activated RTK by determination of the phosphorylation status of MET is more important. Phosphorylation and thereby activation can also occur if MET is not overexpressed; due to mutation or the activation of MET by secreted HGF.

In conclusion, investigation just of one receptor independently of the quantification method might be extended to other RTKs, maybe the focus should not be only on the expression level of RTKs, but on the determination of the activation status.

The results of this study might have consequential clinical implication for therapeutic administration of MET specific drugs, in particular for MET targeting TKIs, namely, that not the expression alone might be of therapeutical relevance, but also the determination of the phosphorylation status.
